# Long-read sequencing identifies *FGF14* repeat expansions in Parkinson’s disease

**DOI:** 10.1093/brain/awaf456

**Published:** 2025-12-02

**Authors:** Fulya Akçimen, Kensuke Daida, Lara M Lange, Abraham Moller, Abigail Miano-Burkhardt, Laksh Malik, Kimberly Paquette, Pilar Alvarez Jerez, Jackson Mingle, Breeana Baker, Melissa Meredith, Cedric Kouam, Paige Jarreau, Androo Markham, Jessica Anderson, Miten Jain, Mark Chaisson, Mark Cookson, Bradford Casey, Hirotaka Iwaki, Sara Bandres-Ciga, Paula Saffie-Awad, Mike A Nalls, Zih-Hua Fang, Andrew B Singleton, Cornelis Blauwendraat, Kimberley J Billingsley

**Affiliations:** Laboratory of Neurogenetics, National Institute on Aging, National Institutes of Health, Bethesda, MD 20892, USA; Laboratory of Neurogenetics, National Institute on Aging, National Institutes of Health, Bethesda, MD 20892, USA; Center for Alzheimer's and Related Dementias, National Institute on Aging and National Institute of Neurological Disorders and Stroke, National Institutes of Health, Bethesda, MD 20892, USA; Laboratory of Neurogenetics, National Institute on Aging, National Institutes of Health, Bethesda, MD 20892, USA; Center for Alzheimer's and Related Dementias, National Institute on Aging and National Institute of Neurological Disorders and Stroke, National Institutes of Health, Bethesda, MD 20892, USA; Laboratory of Neurogenetics, National Institute on Aging, National Institutes of Health, Bethesda, MD 20892, USA; Center for Alzheimer's and Related Dementias, National Institute on Aging and National Institute of Neurological Disorders and Stroke, National Institutes of Health, Bethesda, MD 20892, USA; Center for Alzheimer's and Related Dementias, National Institute on Aging and National Institute of Neurological Disorders and Stroke, National Institutes of Health, Bethesda, MD 20892, USA; Center for Alzheimer's and Related Dementias, National Institute on Aging and National Institute of Neurological Disorders and Stroke, National Institutes of Health, Bethesda, MD 20892, USA; Center for Alzheimer's and Related Dementias, National Institute on Aging and National Institute of Neurological Disorders and Stroke, National Institutes of Health, Bethesda, MD 20892, USA; Center for Alzheimer's and Related Dementias, National Institute on Aging and National Institute of Neurological Disorders and Stroke, National Institutes of Health, Bethesda, MD 20892, USA; DataTecnica LLC, Washington, DC 20037, USA; Center for Alzheimer's and Related Dementias, National Institute on Aging and National Institute of Neurological Disorders and Stroke, National Institutes of Health, Bethesda, MD 20892, USA; Center for Alzheimer's and Related Dementias, National Institute on Aging and National Institute of Neurological Disorders and Stroke, National Institutes of Health, Bethesda, MD 20892, USA; Oxford Nanopore Technologies, OX4 4DQ, UK; Oxford Nanopore Technologies, OX4 4DQ, UK; Department of Bioengineering, Department of Physics, Northeastern University, Boston, MA 02120, USA; Department of Quantitative and Computational Biology, University of Southern California, Los Angeles, CA 90089, USA; Laboratory of Neurogenetics, National Institute on Aging, National Institutes of Health, Bethesda, MD 20892, USA; Department of Clinical Research, The Michael J. Fox Foundation for Parkinson’s Research, New York, NY 10163, USA; Center for Alzheimer's and Related Dementias, National Institute on Aging and National Institute of Neurological Disorders and Stroke, National Institutes of Health, Bethesda, MD 20892, USA; DataTecnica LLC, Washington, DC 20037, USA; Center for Alzheimer's and Related Dementias, National Institute on Aging and National Institute of Neurological Disorders and Stroke, National Institutes of Health, Bethesda, MD 20892, USA; Clínica Santa María, Santiago 7520349, Chile; Department of Specialties, Faculty of Medicine, University of Concepción, Concepción 4070409, Chile; Center for Alzheimer's and Related Dementias, National Institute on Aging and National Institute of Neurological Disorders and Stroke, National Institutes of Health, Bethesda, MD 20892, USA; DataTecnica LLC, Washington, DC 20037, USA; German Center for Neurodegenerative Diseases (DZNE), Tübingen 72076, Germany; Laboratory of Neurogenetics, National Institute on Aging, National Institutes of Health, Bethesda, MD 20892, USA; Center for Alzheimer's and Related Dementias, National Institute on Aging and National Institute of Neurological Disorders and Stroke, National Institutes of Health, Bethesda, MD 20892, USA; Laboratory of Neurogenetics, National Institute on Aging, National Institutes of Health, Bethesda, MD 20892, USA; Center for Alzheimer's and Related Dementias, National Institute on Aging and National Institute of Neurological Disorders and Stroke, National Institutes of Health, Bethesda, MD 20892, USA; Laboratory of Neurogenetics, National Institute on Aging, National Institutes of Health, Bethesda, MD 20892, USA; Center for Alzheimer's and Related Dementias, National Institute on Aging and National Institute of Neurological Disorders and Stroke, National Institutes of Health, Bethesda, MD 20892, USA

**Keywords:** *FGF14*, Parkinson’s disease, short tandem repeats, long-read sequencing

## Abstract

Pathogenic GAA repeat expansions in *FGF14* are an established cause of late-onset cerebellar ataxia, but have not been linked to Parkinson’s disease. Given emerging evidence that repeat expansions in ataxia-associated genes like *RFC1* can contribute to atypical or familial forms of Parkinson’s disease, we investigated whether *FGF14* expansions might play a similar role.

Using long-read whole-genome sequencing, we analysed 411 individuals with Parkinson’s disease and 197 neurologically healthy controls from the Parkinson’s Progression Markers Initiative (PPMI) cohort, together with 1429 additional controls from the National Institutes of Health (NIH) Center for Alzheimer’s Disease and Related Dementias (CARD) initiative, the 1000 Genomes Project, and the All of Us program, representing globally diverse populations.

We identified pathogenic *FGF14* GAA repeat expansions in five individuals with Parkinson’s disease and one control subject. All five individuals fit the clinical criteria of Parkinson’s disease and showed typical patterns of neurodegeneration on DaTSCAN imaging; α-synuclein aggregation was confirmed by a positive seeding assay among four individuals with available data. These findings broaden the phenotypic spectrum of *FGF14* repeat-associated disease and suggest a rare, previously unrecognized genetic contributor to Parkinson’s disease.

To our knowledge, this is the first report implicating *FGF14* in Parkinson’s disease and underscores the utility of long-read sequencing for detecting hidden forms of pathogenic variation in unresolved cases.

## Teaser

Akçimen *et al.* report the first systematic screening for intronic *FGF14* GAA repeat expansions and their association with Parkinson's disease, using long-read sequencing data from over 400 patients and 1600 controls across the PPMI, the All of Us Research Program, the CARD Initiative, and the 1000 Genomes Project.

## Introduction

Most genetic studies of Parkinson’s disease (PD) focused on single-nucleotide variants, while other forms of variation, such as short tandem repeats, are understudied, primarily due to technical limitations of short-read sequencing approaches.^1^ Yet, pathogenic repeat expansions are established causes of several neurological disorders, including frontotemporal dementia, amyotrophic lateral sclerosis and various forms of ataxia.^2^ Interestingly, several repeat expansions primarily linked to other neurological diseases were also observed in patients with PD, including those associated with spinocerebellar ataxia genes, such as *ATXN2* and *ATXN3.*^3,4^ In some PD genetic screening studies, repeat expansions were even observed at unexpectedly high frequencies. Further, biallelic AAGGG repeat expansions in *RFC1*, causing cerebellar ataxia, neuropathy and vestibular areflexia syndrome,^5^ have also been reported in a subset of individuals with PD in European cohorts.^6,7^

Recently, two independent studies identified pathogenic intronic GAA repeat expansions in *FGF14* in patients with late-onset ataxia. These expansions are considered fully penetrant when exceeding 300 repeats [(GAA)_n_ > 300], and partially penetrant in the 250–300 repeat range.^8,9^ While *FGF14* repeat expansions have been investigated in multiple system atrophy–cerebellar type (MSA-C) cohorts, their potential role in PD has not been explored yet.^10-12^

In this study, we screened for pathogenic *FGF14* (GAA)_n_ repeat expansions in individuals with PD by leveraging Oxford Nanopore Technologies (ONT) long-read whole-genome sequencing (WGS) data. Our analysis included 411 PD cases from the Parkinson’s Progression Markers Initiative (PPMI) and 1626 controls obtained from PPMI, the National Institutes of Health (NIH) CARD Long-read initiative, All of Us, and 1000 Genomes Project participants. Additionally, we aimed to characterize repeat motifs in identified carriers, including alleles within the reduced penetrance range (250–300 repeats), and characterize alternative non-pathogenic repeat configurations, including (GAAGGA)_n_, (GAACGA)_n_, and composite motifs such as (GAA)_n_(CGA)_n_.

## Materials and methods

### Cohort information

We obtained frozen blood samples from PPMI (https://www.ppmi-info.org/) and accessed existing long-read WGS from the North American Brain Expression Consortium (NABEC), the Human Brain Collection Core (HBCC), All of Us Research Program release 8 (https://www.researchallofus.org/), and the 1000 Genomes Project (https://ftp.1000genomes.ebi.ac.uk/vol1/ftp/data_collections/1KG_ONT_VIENNA/) datasets. In total, the data comprised 411 PD cases and 1626 neurologically healthy control subjects. All PD cases were diagnosed according to the UK PD Society Brain Bank^13^ criteria. Demographic characteristics of the other cohorts are shown in Supplementary Table 1.

### Long-read sequencing-based screening of *FGF14* repeat expansion

We screened three reference cohorts: (i) 204 European-ancestry controls from the NABEC and 133 African-ancestry controls from HBCC, both generated as part of the CARD Long-Read Initiative; (ii) 908 globally diverse individuals from the 1000 Genomes Long-Read Project; and (iii) 184 European-ancestry healthy controls from the All of Us long-read sequencing dataset.

Sequencing, quality control, alignment, variant calling and methylation analysis for the PPMI dataset are described in detail in the Supplementary material, ‘Methods’ section. Sequencing and downstream processing for the NIH’s CARD Long-read initiative cohorts, specifically the HBCC and NABEC samples, were previously described elsewhere.^14^ In addition, we used long-read WGS data from the All of Us Research Program^15^ and 1000 Genomes Project ONT Panel.^15,16^

The length of the *FGF14* GAA repeat expansion was estimated using *Straglr*, a tool that enables both repeat sizing and the detection of alternative motifs within a defined region.^17^ The repeat locus was defined as chr13:102161576–102161726 (hg38). For each sample, the (GAA) repeat length was calculated as the average of the repeat lengths from the 10 reads with the longest observed expansions. For All of Us samples, where allelic depth was lower, estimates were based on the five longest reads. To assess the presence of interruptions or alternative repeat motifs, we generated waterfall plots using RepeatAnalysis tools (https://github.com/PacificBiosciences/apps-scripts/tree/master/RepeatAnalysisTools).

## Results

### Identifying carriers of the *FGF14* GAA repeat expansions

**Figure 1:**
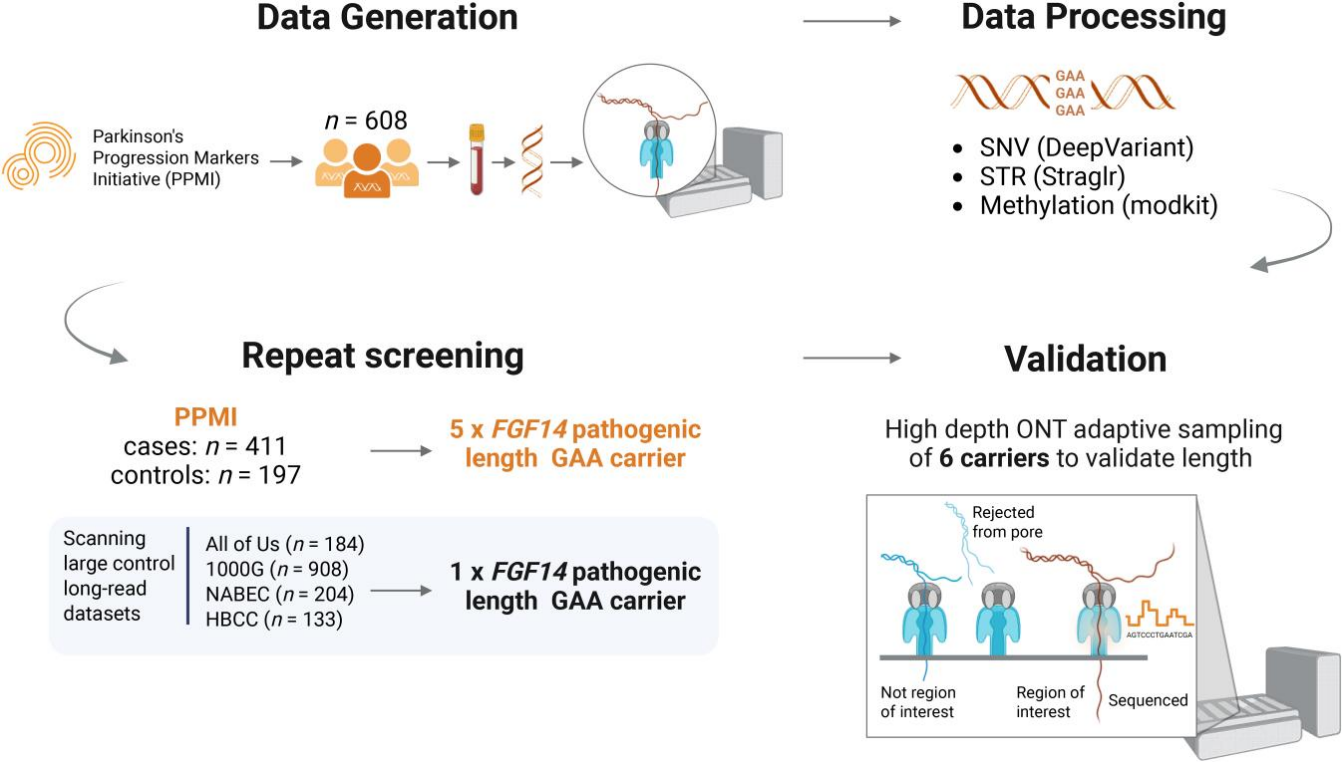
**Study design for *FGF14* repeat expansion screening using long-read sequencing.** Schematic overview of the analytical workflow and study rationale, highlighting the use of ONT sequencing to identify pathogenic GAA repeat expansions in *FGF14*. Created in BioRender Billingsley, K. (2025) https://BioRender.com/lhbt0z3. HBCC = Human Brain Collection Core; NABEC = North American Brain Expression Consortium; ONT = Oxford Nanopore Technologies; PPMI = Parkinson’s Progression Markers Initiative; SNV = single nucleotide variant; STR = short tandem repeat.

We screened for intronic GAA repeat expansions in *FGF14* using ONT long-read WGS in individuals from the PPMI dataset. Sequencing quality metrics, including read N50, median coverage and total data yield, are summarized in Supplementary Table 2. An overview of the study design is presented in Fig. 1.

**Figure 2:**
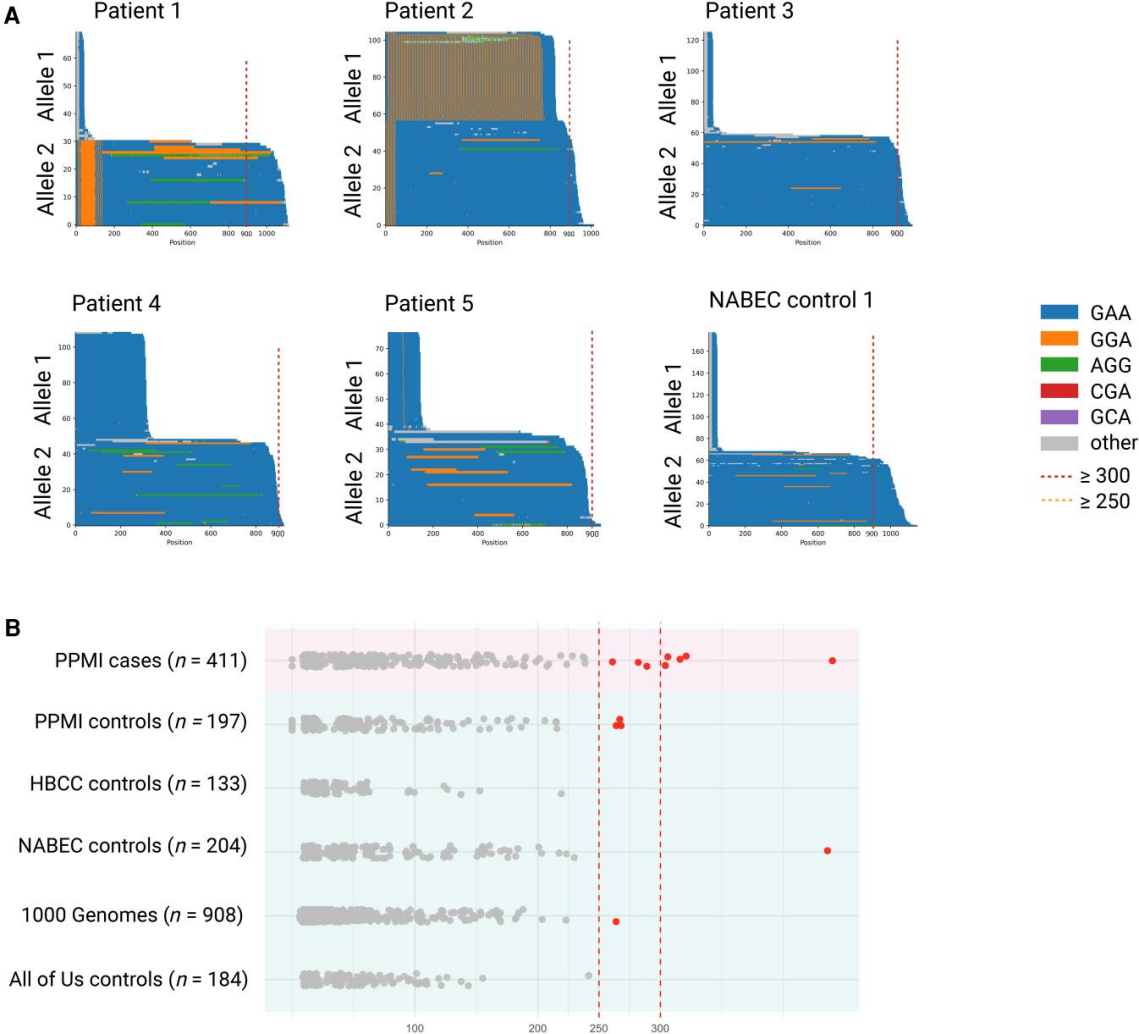
**Detection of Pathogenic *FGF14* (GAA)_n_ repeat expansions in Parkinson’s disease cases. (A)** Waterfall plots displaying the repeat lengths observed in five Parkinson’s disease (PD) cases carrying fully penetrant (GAA)_n_ repeat expansion ≥300 repeat units, as determined by adaptive sampling long-read sequencing. (**B)** Swimlane plot showing the distribution of *FGF14* (GAA)_n_ repeat lengths in the PPMI cases (*n* = 411), the PPMI controls (*n* = 197), in the NABEC/HBCC control cohorts (*n* = 317) comprising individuals of European and African and African-admixed ancestry, the 1000 Genomes Project control cohort (*n* = 908) comprising individuals of mixed ancestry, and the All of Us biobank participants (*n* = 184) comprising individuals of European ancestry. Dashed red vertical lines denote the thresholds for reduced penetrance (250 repeat units) and full penetrance (300 repeat units), respectively. HBCC = Human Brain Collection Core; NABEC = North American Brain Expression Consortium PPMI = Parkinson’s Progression Markers Initiative.

In the PPMI cohort, we identified five patients with PD of European ancestry carrying *FGF14* GAA expansions above the pathogenic threshold of 300 GAA repeats (Fig. 2), while no carriers were identified in the PPMI controls. Except for one individual (Patient 1, who carried the *GBA1* p.Leu483Pro variant), these expansions were not accompanied by known pathogenic variants in established genes linked to PD [including *GBA1*, *LRRK2*, *SNCA*, *PARK7*, *FBXO7*, *PINK1*, *PRKN*, *PLA2G6*, *VPS13C* and *VPS35*]. Among the control reference datasets, we identified one individual of European ancestry from the NABEC control brain dataset carrying an *FGF14* GAA expansion longer than 300 GAA repeats, exceeding the pathogenic threshold. This was a male individual who died at 80 years of age without any reported neurological symptoms. All six individuals, including the five PPMI PD patients and the NABEC control, were further validated by adaptive sampling long-read sequencing. The average depth of coverage over the *FGF14* repeat region, based on adaptive sampling long-read sequencing, was 55×, 125×, 90×, 97× and 117× for PPMI Patients 1–5, respectively, and 114× in the NABEC control (Fig. 2).

A summary of *FGF14* repeat lengths, including the presence of alternative motifs in the repeat region, is provided for a total of 411 PD cases and 1626 controls across all investigated cohorts in Supplementary Table 2. Our analysis in a subset of 386 PD cases and 722 controls of European ancestry show that carrying uninterrupted *FGF14* GAA expansions of ≥300 repeats is associated with PD (Fisher’s exact test; *P* = 0.022), while having ≥250 repeats is not associated with disease risk (Fisher’s exact test; *P* = 0.31). Repeat length modelled as a continuous variable was also nominally associated with PD risk [odds ratio (OR) = 1.003 per repeat unit, 95% confidence interval (CI: 1.000–1.005), *P* = 0.015].

We further investigated a potential correlation between repeat length and age at onset, as reported by previous studies in ataxia cohorts. Among the five identified *FGF14* expansion carriers, ages at onset ranged from 37 to 56 years. There was no correlation between repeat length and age at onset (*R*^2^ = 0.02, *P* = 0.85; Supplementary Fig. 3). In addition, to examine whether increased repeat length is associated with earlier age at onset among patients, we performed a linear regression analysis. No significant associations were found between repeat length and age at onset (*R*^2^ = 0.01, *P* = 0.06; Supplementary Fig. 3).

To interpret these findings, we examined *FGF14* repeat length variation across several large long-read control datasets. In these datasets, uninterrupted *FGF14* GAA repeat lengths ranged from 9 to 436 in NABEC, 10 to 219 in HBCC, 9 to 242 in All of Us, and 6 to 264 in 1000 Genomes samples (Fig. 2 and Supplementary Table 2). These results define the normal range of *FGF14* repeat length across ancestrally diverse control populations and provide a critical reference framework for interpreting expansions in patient cohorts. Collectively, our findings reinforce the pathogenic size threshold of ≥300 repeats and establish a foundation for future studies investigating the role of *FGF14* in neurodegenerative diseases.

*FGF14* locus was hypermethylated in blood across all samples from PPMI, while brain-derived DNA from the cerebellum of the NABEC carrier showed hypomethylation without allele-specific differences (Supplementary Fig. 4).

### Characterization of *FGF14* repeat motif structure in Parkinson’s disease

We identified seven individuals, three patients with PD and four controls (three from the PPMI cohort and one from the 1000 Genomes Project), carrying *FGF14* (GAA)_n_ repeat expansions in the reduced penetrance range of 250–300 repeats. In addition to pure GAA expansions, both Rafehi *et al*.^9^ and Pellerin *et al*.^8^ reported complex or interrupted motifs, such as composite structures involving (GAAGGA)_n_ or [(GAA)4(GCA)1]_n_, raising the possibility that sequence composition, not just repeat length, may influence pathogenicity.^18^ To investigate whether similar motif variability is present in PD, we characterized the repeat structure at the *FGF14* locus in ONT long-read WGS data from fully penetrant and reduced penetrance expansion carriers in cases and control cohorts. We identified 11 controls carrying the (GAAGGA)_n_ motif, two from the PPMI cohort, four from NABEC, three from the 1000 Genomes Project [including one European (Utah residents with Northern and Western European ancestry), one American (Puerto Rican in Puerto Rico) and one South Asian ancestry (Sri Lankan Tamil in the UK)] and two from the All of Us European ancestry cohort. Additionally, we identified one PPMI case and 13 controls from the 1000 Genomes Project carrying the (GAAGCA)_n_ motif, six of East Asian and seven of American ancestry, as well as a composite (GAA)_n_(GCA)_n_ motif in a 1000 Genomes participant of East Asian ancestry.

### Clinical features of the Parkinson's disease patient carrying the pathogenic *FGF14* GAA expansion

The clinical features of all identified carriers are summarized in Table 1 and are reported in detail in the following sections.

**Table 1:** Clinical characteristics of the patients with PD carrying a pathogenic length *FGF14* GAA expansion

Individual ID	Individual 1	Individual 2	Individual 3	Individual 4	Individual 5
*FGF14* (GAA)_n_ expansion length	440	321	316	306	304
**Basic demographic and clinical characteristics**					
Ancestry	EUR	EUR	EUR	EUR	EUR
Gender	Male	Female	Male	Female	Male
Diagnosis	PD	PD	PD	PD	PD
Age at baseline	50	57	39	51	48
Age at onset	49	56	37	50	45
Age at diagnosis	50	57	38	51	47
Age at most recent assessment	63	64	41	61	60
Disease duration/years of follow-up	13	7	2	10	12
Family history of PD	Father with PD	No	No	No	No
**PD motor and non-motor features**					
Bradykinesia	+	+	+	+	+
Tremor	−	+	+	−	+
Rigidity	+	+	+	+	+
Postural instability	+	−	−	−	−
RBD	+	−	−	-	+
Hyposmia	+	+	−	+	+
MCI	+	−	−	−	−
Autonomic features	URIN, CNST, LTHD	URIN	−	CNST	URIN
Neuropsychiatric features	DPRS, ANXS, APAT	−	−	DPRS, ANXS	−
UPDRS I score	27	6	2	9	12
UPDRS II score	28	6	2	11	16
UPDRS III score	NA	19	23	NA	61 (59)
UPDRS III score ON	63	18	23	24	45 (59)
UPDRS IV score	12	0	NA	0	0 (59)
Hoehn and Yahr score	NA	2	1	NA	2 (59)
Hoehn and Yahr score ON	2	2	1	1	2 (59)
LEDD	1650.4	310	0	890	709.5
**Diagnostics**					
DaTSCAN, mean caudate	1.3399 (54)	1.395 (61)	1.815 (40)	1.705 (55)	1.1 (52)
DaTSCAN, mean putamen	0.445 (54)	0.435 (61)	0.825 (40)	0.85 (55)	0.445 (52)
DaTSCAN, mean striatum	0.8925 (54)	0.915 (61)	1.3199 (40)	1.2775 (55)	0.7725 (52)
MRI	No cerebellar atrophy	NA	No cerebellar atrophy	No cerebellar atrophy	No cerebellar atrophy
SAA (CSF)	+	+	+	+	NA
Other genetic findings^a^	*GBA1* p.Leu483Pro	−	−	−	−
* APOE* haplotype	E2/E3	E2/E3	E3/E3	E3/E3	E3/E3

Symptoms were considered present if rated as slight (score ≥1) or higher on the corresponding scale. Symptom status reflects the most recent assessment; ages in parentheses indicate the time of the last available evaluation if not assessed at the latest visit. (+/−) = present/absent; ANXS = anxiety; APAT = apathy; CNST = constipation; DPRS = depression/depressive symptoms; EUR = European ancestry; LEDD = levodopa equivalent daily dose; LTHD = light-headedness; NA = not available; PD = Parkinson's disease; SAA = α-synuclein seed amplification assay; URIN = uninary problems.

^a^Other genetic findings only include known variants in GBA1, LRRK2 and SNCA.

#### Individual 1

This 63-year-old male had an age at motor symptom onset (AAO) of 49 years. Initial motor symptoms included mild unilateral bradykinesia and rigidity [Unified Parkinson’s Disease rating Scale (UPDRS) III, 16 points; Hoehn and Yahr score, 1; untreated). He also reported mild non-motor signs, including constipation, light-headedness, fatigue, pain, depression and sleep impairment (UPDRS I, 8 points). Levodopa was initiated 3 years after onset; the levodopa-equivalent daily dose (LEDD) at his latest follow-up after 13 years of disease was 1650 mg. Over the disease course, symptoms progressed to bilateral motor involvement, gait impairment with slight freezing, postural instability (UPDRS III, 43 points; Hoehn and Yahr score 2; ON stage), and motor complications, including dyskinesias and painful off-dystonia. He had mild cognitive impairment (Montreal Cognitive Assessment, 24/30 points at age 60) and hyposmia. The α-synuclein seed amplification assay (SAA) was positive, and the DaTSCAN showed reduced putaminal uptake with relative caudate sparing, consistent with neurodegenerative PD. MRI did not show cerebellar atrophy. Notably, this individual had a positive family history of PD with an affected father, and he also carried the *GBA1* p.Leu483Pro variant.

#### Individual 2

This individual is a 64-year-old female with PD onset at age 56. Her initial motor symptoms were mild unilateral bradykinesia, rigidity and tremor (UPDRS III, 17 points; Hoehn and Yahr score, 1; untreated). For non-motor signs, she reported mild constipation and urinary problems (UPDRS I, 3 points). She also has hyposmia. She was started on levodopa in the first year after onset, but only required small dosages (LEDD 310 mg at her latest assessment after 7 years of disease duration). Over the course of the disease, she developed slight bilateral motor involvement, but her symptoms remained fairly stable otherwise (UPDRS III, 18 points; Hoehn and Yahr score, 2; ON stage). SAA was positive, and the DaTSCAN showed reduced putaminal uptake with relative caudate sparing, consistent with neurodegenerative PD. No MRI was performed for this individual.

#### Individual 3

The 41-year-old male developed first PD symptoms at age 37. Initial motor symptoms were mild unilateral bradykinesia, rigidity and tremor (UPDRS III, 14 points; Hoehn and Yahr score 1; untreated). Non-motor signs were mild and included constipation and sleep impairment (UPDRS I, 4 points). He remained untreated over a disease course of 2 years, while his motor symptoms progressed but remained unilateral (UPDRS III, 23 points; Hoehn and Yahr score, 2; ON stage). He had no hyposmia. SAA was positive, and the DaTSCAN showed findings suggestive of early dopaminergic degeneration. MRI did not show cerebellar atrophy.

#### Individual 4

This 61-year-old female had PD onset at age 50. Her initial motor symptoms were mild unilateral bradykinesia and rigidity (UPDRS III, 15 points; Hoehn and Yahr score, 1; untreated). Levodopa was initiated in the first year after onset, with a moderate LEDD of 890 mg at her latest assessment after 10 years of disease. Over the course of the disease, she only showed mild motor progression (UPDRS III, 24 points; Hoehn and Yahr score, 1; ON stage) but reported several non-motor signs, including anxiety and depressive episodes, fatigue, pain and constipation. She also had hyposmia. SAA was positive, and the DaTSCAN showed mildly reduced putaminal uptake with preserved caudate, potentially indicating early or mild dopaminergic dysfunction. MRI did not show cerebellar atrophy.

#### Individual 5

The 60-year-old male had an AAO of 45 years. Initial symptoms included mild unilateral bradykinesia, rigidity, and tremor (UPDRS III, 12 points; Hoehn and Yahr score, 1; untreated); no non-motor signs except hyposmia (UPDRS I, 0 points). Levodopa was initiated 3 years after onset; the LEDD at his last follow-up after a 12-years disease course was 709 mg. Over 12 years of follow-up, he had a progressive disease course with bilateral motor involvement without relevant motor complications (UPDRS III, 36 points; Hoehn and Yahr score, 2; ON stage). The DaTSCAN showed reduced putaminal uptake with relative caudate sparing, consistent with neurodegenerative PD; SAA was not available. MRI did not show cerebellar atrophy.

## Discussion

In this study, we report the first systematic screening of intronic *FGF14* GAA repeat expansions in PD, leveraging long-read WGS data from 463 patients with PD and 1627 control subjects from PPMI, the All of Us Research Program, the CARD Initiative and the 1000 Genomes Project. We identified five PD patients and one control carrying pathogenic length *FGF14* GAA expansions. A similar frequency in controls was previously reported by Mohren *et al*.,^19^ who identified 2 of 802 controls carrying pathogenic *FGF14* GAA expansions, supporting the notion of incomplete penetrance within this repeat size range. We also assessed alleles within the reduced penetrance range (250–300 repeats) and characterized alternative, non-pathogenic repeat configurations, including non-pathogenic (GAAGGA)_n_, (GAACGA)_n_, and composite motifs such as (GAA)_n_(CGA)_n_, providing what is, to our knowledge, the most comprehensive population-level analysis to date.

The clinical profiles of the five individuals carrying pathogenic *FGF14* repeat expansions were consistent with PD without pronounced atypical features. While most individuals had a rather mild to moderate disease course, one individual showed marked progression with motor complications and development of mild cognitive impairment. Notably, this individual also carried the *GBA1* p.Leu483Pro variant in addition to a pathogenic *FGF14* repeat expansion. This individual also was the only one with a positive family history of PD. Interestingly, four individuals had an early AAO ≤50 years, with AAO overall ranging from 37 to 56 years; however, there was no correlation between *FGF14* repeat length and AAO. Variable non-motor features were reported, but were generally mild; hyposmia was reported in four individuals. SAA was positive for all four tested individuals, and DaTSCAN imaging consistently demonstrated findings suggestive of neurodegenerative PD. Notably, the clinical assessment was tailored to PD, and possible (mild) cerebellar signs, typical for *FGF14* repeat expansion carriers, may not have been adequately assessed. However, none of the four individuals with available MRI had cerebellar atrophy. While one individual carried a *GBA1* variant, none of the identified *FGF14* repeat expansion carriers harboured a disease-explaining genetic variant in a gene linked to monogenic PD.

Our findings should be interpreted in the context of several limitations. First, the current analysis was restricted to individuals of European ancestry. Future studies in diverse populations are needed to better define the global frequency and phenotypic impact of *FGF14* repeat expansions in PD. Second, we observed that the *FGF14* locus was hypermethylated in blood-derived DNA across both carriers and non-carriers, while cerebellum-derived DNA from the NABEC carrier showed relative hypomethylation without allele-specific differences (Supplementary Fig. 4). These findings are consistent with existing literature showing that *FGF14*’s low expression in blood and higher expression in the brain; however, they are based on a single cerebellum sample and should be interpreted with caution. Third, although a pathogenic threshold of ≥300 GAA repeats has been proposed based on prior work in ataxia, the penetrance, expressivity and mechanistic impact of these expansions in the context of PD remain to be fully defined.

Taken together, our results suggest that pathogenic *FGF14* GAA repeat expansions are present among PD patients of European descent, with an estimated frequency of 1.22%. While we identified five carriers of fully penetrant *FGF14* repeat expansions, one of whom also carried a *GBA1* variant, the clinical significance in the context of PD remains unknown. Follow-up analyses in larger and more diverse PD cohorts, including segregation and functional studies, are required to determine whether this genetic variation plays a causal role in PD or represents a coincidental finding without disease relevance. Integrative analyses incorporating long-read sequencing, transcriptomics, and methylation profiling from brain tissue will be essential to determine the functional relevance of *FGF14* expansions in PD and other neurodegenerative disorders.

## Supplementary Material

awaf456_Supplementary_Data

## Data Availability

Extracted DNA for 1000 Genomes Project was obtained from the Coriell Institute for Medical Research and was consented for the full public release of genomic data. Please see Coriell (https://www.coriell.org) for more information on specific cell lines. 1000 Genomes Project ONT dataset was generated at the Institute of Molecular Pathology (Vienna, Austria) with funds provided by Boehringer-Ingelheim. All of Us genomic data are publicly available to registered researchers on the All of Us Researcher Workbench at https://www.researchallofus.org/data-tools/workbench/. Researchers can apply for access to the All of Us database following the instructions at https://www.researchallofus.org/register/. PPMI data used in the preparation of this article were obtained on 2025-06-01 from the PPMI database (www.ppmi-info.org/access-data-specimens/download-data), RRID: SCR_006431. For up-to-date information on the study, visit www.ppmi-info.org. The PPMI ONT data will be available at the LONI IDA. The pipeline and analyses presented in this manuscript are publicly available at https://github.com/NIH-CARD/CARDlongread_FGF14_repeat_expansion.
