# Supplementary material for: Long-read sequencing identifies FGF14 repeat expansions in Parkinson’s disease
Source: Brain. 2025 Dec 2;149(5):1514–21. doi: 10.1093/brain/awaf456 (PMC13140659; doi:10.1093/brain/awaf456)
Supplement: awaf456_Supplementary_Data [file awaf456_supplementary_data.zip › brain-2025-02349-File006.pdf]

Supplementary Figures

Supplementary Figure 1. Integrative Genomics Viewer visualization of the *FGF14* $(GAA)_n$ repeat expansion in affected carriers.

Genome browser snapshots showing aligned long-read sequencing reads at the *FGF14* locus for the five PD patients carrying the pathogenic expansion. Expanded alleles are indicated by increased repeat length relative to the reference.

Patient 1

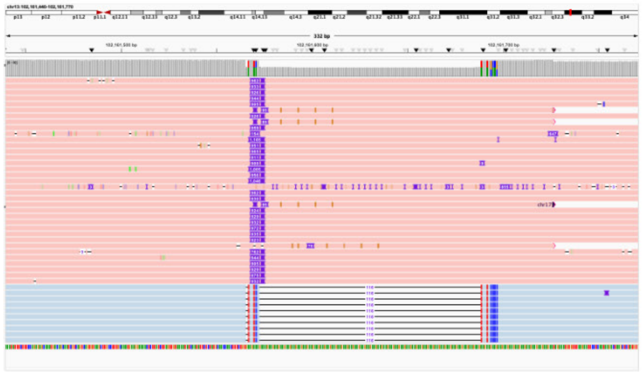

Patient 2

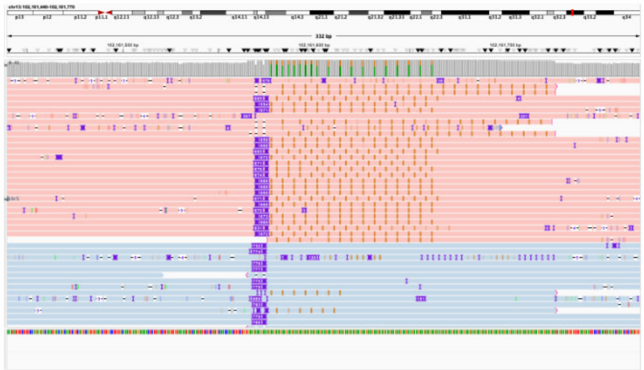

Patient 3

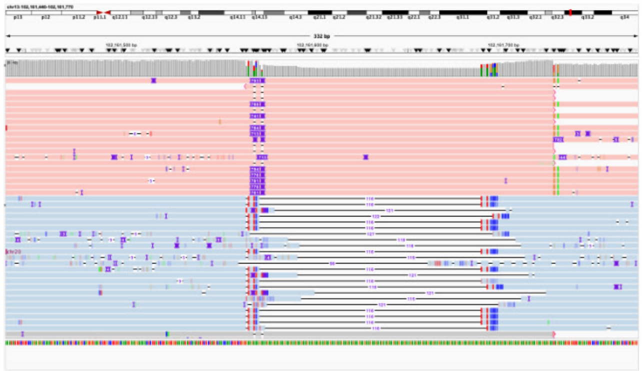

Patient 4

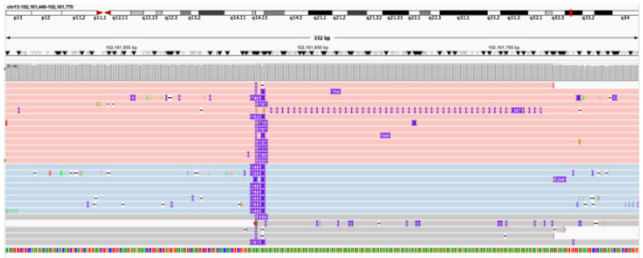

Patient 5

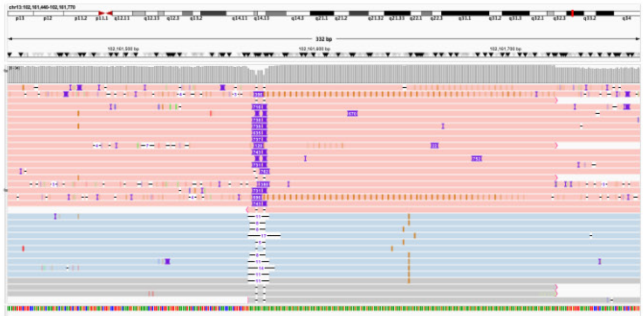

NABEC control 1

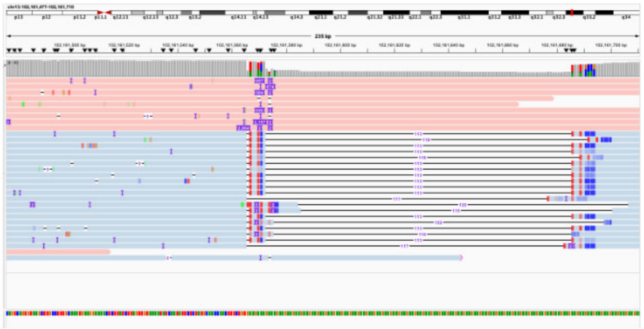

Supplementary Figure 2. Pathogenic and reduced penetrance *FGF14* $(GAA)_n$ repeat expansions identified in PPMI Parkinson's disease patients. a) Waterfall plot displaying the repeat lengths observed in five Parkinson's disease cases carrying fully penetrant $(GAA)_n$ expansion ≥300 repeat units, **b)** three patients and three healthy controls with reduced penetrance *FGF14* $(GAA)_n$ ≥ 250 repeat units, **c)** one patient with $(GAAGCA)_n$ motif, **d)** two controls with $(GAAGGA)_n$ expansions.

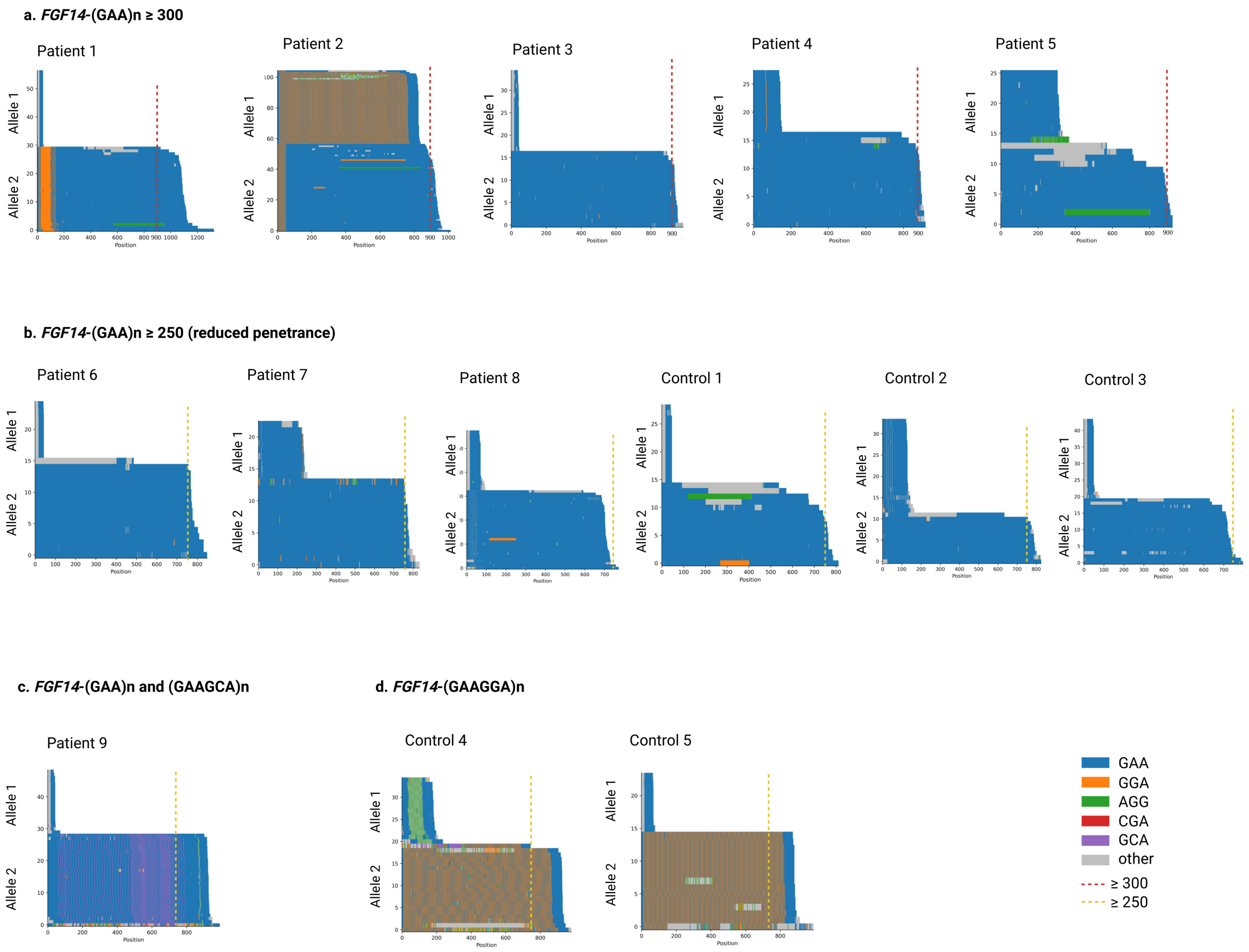
a. FGF14-(GAA)n ≥ 300
Patient 1
Patient 2
Patient 3
Patient 4
Patient 5
b. FGF14-(GAA)n ≥ 250 (reduced penetrance)
Patient 6
Patient 7
Patient 8
Control 1
Control 2
Control 3
c. FGF14-(GAA)n and (GAAGCA)n
Patient 9
d. FGF14-(GAAGGA)n
Control 4
Control 5
Allele 1
Allele 2
Position
GAA
GGA
AGG
CGA
GCA
other
≥ 300
≥ 250

Supplementary Figure 3. Distribution of age at onset by *FGF14* $(GAA)_n$ repeat length. Analysis of five *FGF14*-GAA expansion carriers from the PPMI cohort (left), and analysis of all PPMI Parkinson's disease cases with available age at onset and repeat length data (right).

Supplementary Figure 3

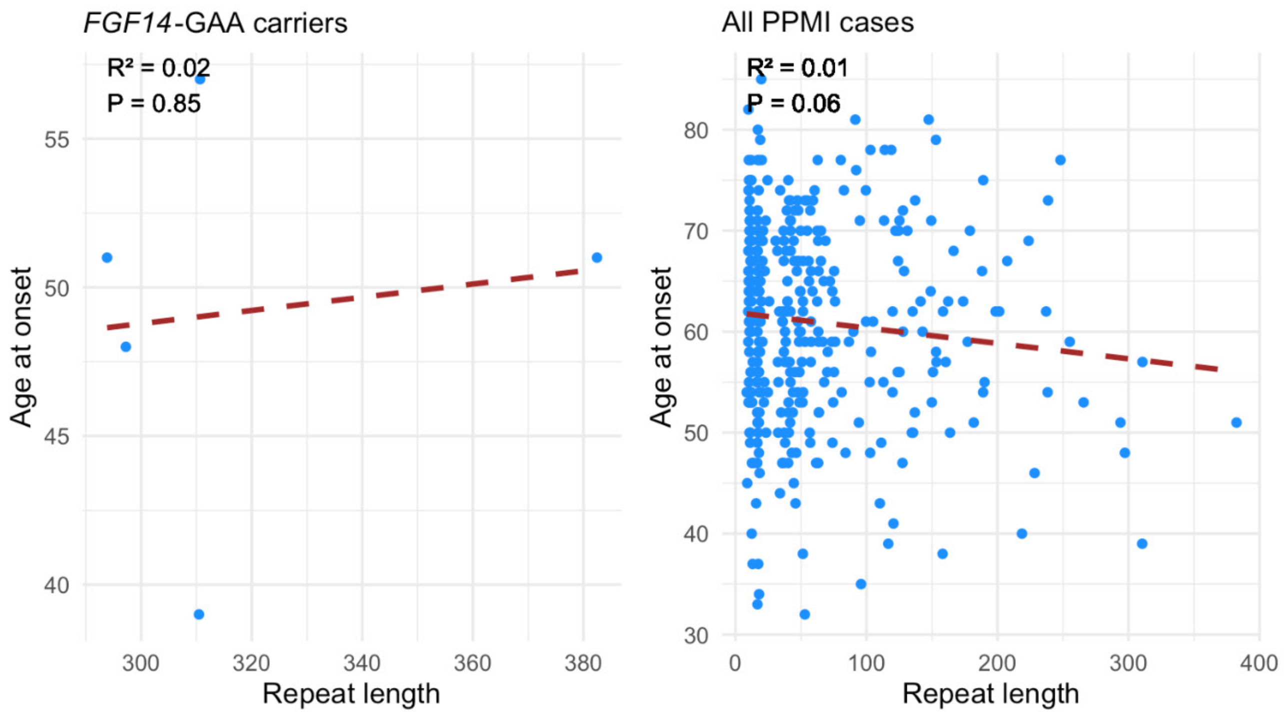

Supplementary Figure 4. Haplotype-specific DNA methylation across the *FGF14* $(GAA)_n$ expansion a–e) Methylation frequency plots generated with modbamtools for PD patients heterozygous for a pathogenic *FGF14* $(GAA)_n$ expansion, based on PPMI blood-derived long-read sequencing data. **f)** Methylation frequency plot for the control individual from the NABEC cohort, based on adaptive sampling from cerebellum tissue. Haplotypes are phased, with haplotype 1 corresponding to the non-expanded allele and haplotype 2 representing the expanded allele. Methylation frequency is shown above, with the *FGF14* gene structure overlaid. Individual reads are shown below, with blue indicating hypomethylation and red indicating hypermethylation.

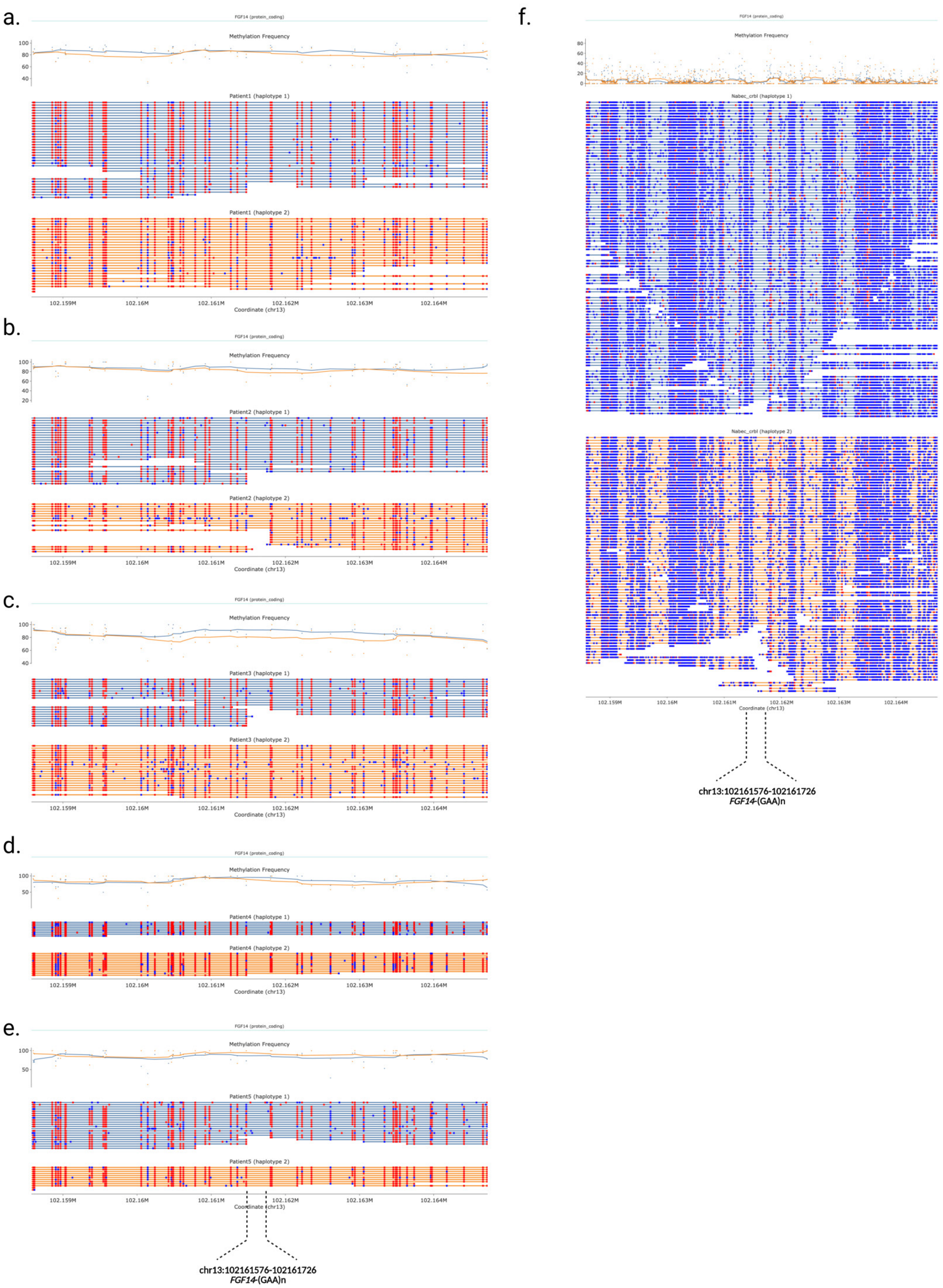
a.
FGF14 (protein_coding)
Methylation Frequency
Patient1 (haplotype 1)
Patient1 (haplotype 2)
102.159M
102.16M
102.161M
102.162M
102.163M
102.164M
Coordinate (chr13)
b.
FGF14 (protein_coding)
Methylation Frequency
Patient2 (haplotype 1)
Patient2 (haplotype 2)
Coordinate (chr13)
c.
FGF14 (protein_coding)
Methylation Frequency
Patient3 (haplotype 1)
Patient3 (haplotype 2)
Coordinate (chr13)
d.
FGF14 (protein_coding)
Methylation Frequency
Patient4 (haplotype 1)
Patient4 (haplotype 2)
Coordinate (chr13)
e.
FGF14 (protein_coding)
Methylation Frequency
Patient5 (haplotype 1)
Patient5 (haplotype 2)
Coordinate (chr13)
chr13:102161576-102161726
FGF14-(GAA)n
f.
FGF14 (protein_coding)
Methylation Frequency
Nabec_crbl (haplotype 1)
Nabec_crbl (haplotype 2)
Coordinate (chr13)
chr13:102161576-102161726
FGF14-(GAA)n
